# How Does the Phasianidae Maintain Its Diversity in Central China?

**DOI:** 10.1002/ece3.73796

**Published:** 2026-06-15

**Authors:** Qian Li, Tengwei Su, Yunchuan Zha, Zhaoyuan Li

**Affiliations:** ^1^ Southwest Survey and Planning Institute of National Forestry and Grassland Administration Kunming China; ^2^ School of Forestry Yunnan Forestry Technological College Kunming China; ^3^ College of Soil and Water Conservation Southwest Forestry University Kunming China

**Keywords:** community ecology, evolution, network analysis, Phasianidae, spatial separation, speciation

## Abstract

The hypothesis of allopatric speciation suggests that spatial separation is the major driver to speciation. The ecological niche theory suggests that differentiations in niche dimensions allow more species to co‐exist in ecological communities. It is thus predicted that, (1) species from the same genus will tend to appear in different ecological communities due to the strong force of exclusion between them; (2) sibling genera may co‐occur in communities without direct spatial associations (i.e., with indirect spatial associations); and (3) when they are directly associated, the genera will be different in some ecological features to avoid resource competitions. In this study, we used infrared camera trap data and statistic techniques to set up species association networks in four national nature reserves (NNRs) in central China, and the data of mitochondrial cytochrome b (cytb) gene sequences downloaded from the National Center for Biotechnology Information (NCBI) database to test the predictions based on the phasianids (order Galliformes) appearing in the networks. We found 14 phasianid species belonging to 12 genera, resulting in 91 potential species pairs and 45 potential genus pairs. A total of 12 associations were identified between species, accounting for 13.2% of the potential species pairs; and 11 associations identified between genera, accounting for 24.4% of the potential genus pairs. The chi‐square goodness‐of‐fit test results showed that the spatial separation existed extensively between the phasianids at levels of species (86.8% of potential species pairs) and genera (75.6% of potential genus pairs). There were 2 genera each containing 2 species; i.e., the *Crossoptilon* (containing the white‐eared pheasant *C. crossoptilon* and the blue‐eared pheasant *C. auritum* ) and the *Chrysolophus* (the golden pheasant *C. pictus* and the Lady Amherst's pheasant *C. amherstiae* ). These congeneric species appeared in different ecological communities. The other 10 genera each contained only one species in community, although they have more species in taxonomy. This monotypicalness in community makes them effectively avoid competition within genera. The Spearman rank‐order correlation coefficient tests showed no general correlation between the phylogenetic distance and the spatial association coefficient of the genera of Phasianidae, but the correlation existed in Liancheng, which may be attributed to the difference in evolutionary history between Palearctic and Indomalayan realms. Discussions suggested that the genus pairs of *Tetraophasis*‐*Ithaginis* and *Ithaginis*‐*Crossoptilon* are associated in a way that is consistent with beneficial interactions. The genera in symmetric associations may differentiate in diets. It is thus concluded that the spatial separation may play a major role in promoting and maintaining the diversity of Phasianidae, and the differentiation in ecological features play a supplementary role.

## Abstract 2

Allopatric speciation and ecological niche theory predict that closely related phasianid taxa should exhibit spatial separation, with co‐occurring genera differing ecologically to avoid competition. Using camera‐trap data and mitochondrial cytb sequences across four Chinese national nature reserves, we found extensive spatial separation among phasianids species and genera, congeneric species occurring in distinct communities, and no general correlation between phylogenetic distance and spatial association, though beneficial or noncompetitive interactions arose among some ecologically differentiated genera. We conclude that spatial separation is the primary driver of phasianid diversity, with ecological differentiation acting as a secondary mechanism.

## 1 Introduction

The allopatric speciation hypothesis (Mayr 1947) suggests that local populations will be faced with different pressure, which promotes accumulation of new features in different populations. These populations will eventually evolve into new species (Schluter 2000; Rundle and Nosil 2005). Because of the short evolutionary time, newly evolved species are highly similar in biological characteristics, including ecological habits. Therefore, when encountering again, they will be faced with intense competition, which is detrimental to the development of the species diversity of the clade (Gause's principle; Gause 1936; Futuyma 2013; Heams et al. 2017), and spatial separation between the species will protect them from intense competition (the hypothesis of divergent evolution; Futuyma 2013). It is thus predicted that the extant species of an evolutionarily successful taxon should keep spatially separated from each other. The newly evolved species compose a genus. The genera from the same ancestor will be faced with choices of further evolutionary routes. (1) They keep spatially diverging from each other and occupy more and more geographic spaces, so as to avoid competition. It is thus predicted that the phylogenetic distance is negatively correlated to the strength of spatial association between them; i.e., the greater the distance is, the weaker the association is. This route will lead to low diversity of their clade, because further addition of species into ecological communities is halted by spatial saturation. (2) The genera evolve new differentiations in ecological requirements, such as diets or activity time, which alleviates their competition when they re‐meet again, because they obtain different resources although co‐occurring in space. This route will allow more species to be packed into an ecological community and lead to high diversity of the clade. It is thus predicted that the phylogenetic distance is not correlated to the strength of spatial association, because the spatial co‐occurrence does not necessarily mean severe competition between the genera.

The above predictions have not been tested until recently due to lack of proper data collection techniques and data processing methodology. For exploring the structure of ecological communities, we collected data in Liancheng National Nature Reserve (NNR hereafter) in Gansu Province, China, and Tangjiahe, Wolong, and Heizhugou NNRs in Sichuan Province from 2017 to 2021 using infrared camera traps, and used the statistical techniques of Phi and Lambda coefficients to set up spatial associations of endothermal terrestrial animal species. With this methodology, we were able to show species networks and analyze the possible interactions of takins ( *Budorcas taxicolor* ; Yang et al. 2021), snow leopards ( *Panthera uncia* ; Zhou et al. 2021), sambars ( *Rusa unicolor* ; Q. Li et al. 2022), Sichuan snub‐nosed monkeys ( *Rhinopithecus roxellana* ; Liu et al. 2022; Wang et al. 2023), giant pandas (*Ailuropoda melanoleuca*; Liu et al. 2023), and Tibetan macaques ( *Macaca thibetana* ; Li et al. 2024) with other species that may underlie their spatial associations. To test the above prediction, we analyzed the spatial relationship of mustelids, an evolutionary successful mammal family, and concluded that spatial segregation may serve as a biogeographic mechanism to maintain and promote the species diversity of the family Mustelidae (Liu et al. 2024).

The Phasianidae, an avian family of the order Galliformes, includes 54 genera and 187 species (Gill et al. 2025). This family is divided into 3 subfamilies: Phasianinae, Coturnicinae, and Arborophilinae (Chen et al. 2021; Kimball et al. 2021; Zheng 2015), and is widely distributed across all continents except for Antarctica. A total of 21 genera and 34 species of birds are found in China in a variety of habitats ranging from tundra to tropical rainforests, and from high mountains to lakes and wetlands (Zheng 2015, 2023), indicating that the family is an evolutionarily successful taxon. Yao and colleagues explore the phylogenetic structure of phasianids in China in traditional way without considering ecological context (Yao et al. 2022). However, his earlier comparison on the habitat selection of snow partridges ( *Lerwa lerwa* ) and Tibetan snowcocks ( *Tetraogallus tibetanus* ) shows that the two phasianid species have evolved adaptations to different temperature environments and thus avoided competition (Yao 2017). This finding is similar to those we obtained from our work on mustelids (Liu et al. 2024). In this paper, we used our infrared camera trap data on phasianids from the above four NNRs and the data of mitochondrial cytochrome b (cytb) gene sequences we downloaded from the National Center for Biotechnology Information (NCBI) database (https://www.ncbi.nlm.nih.gov) to test the above predictions to further verify the evolutionary hypotheses mentioned above.

## 2 Methods

### 2.1 Study Area

**FIGURE 1:**
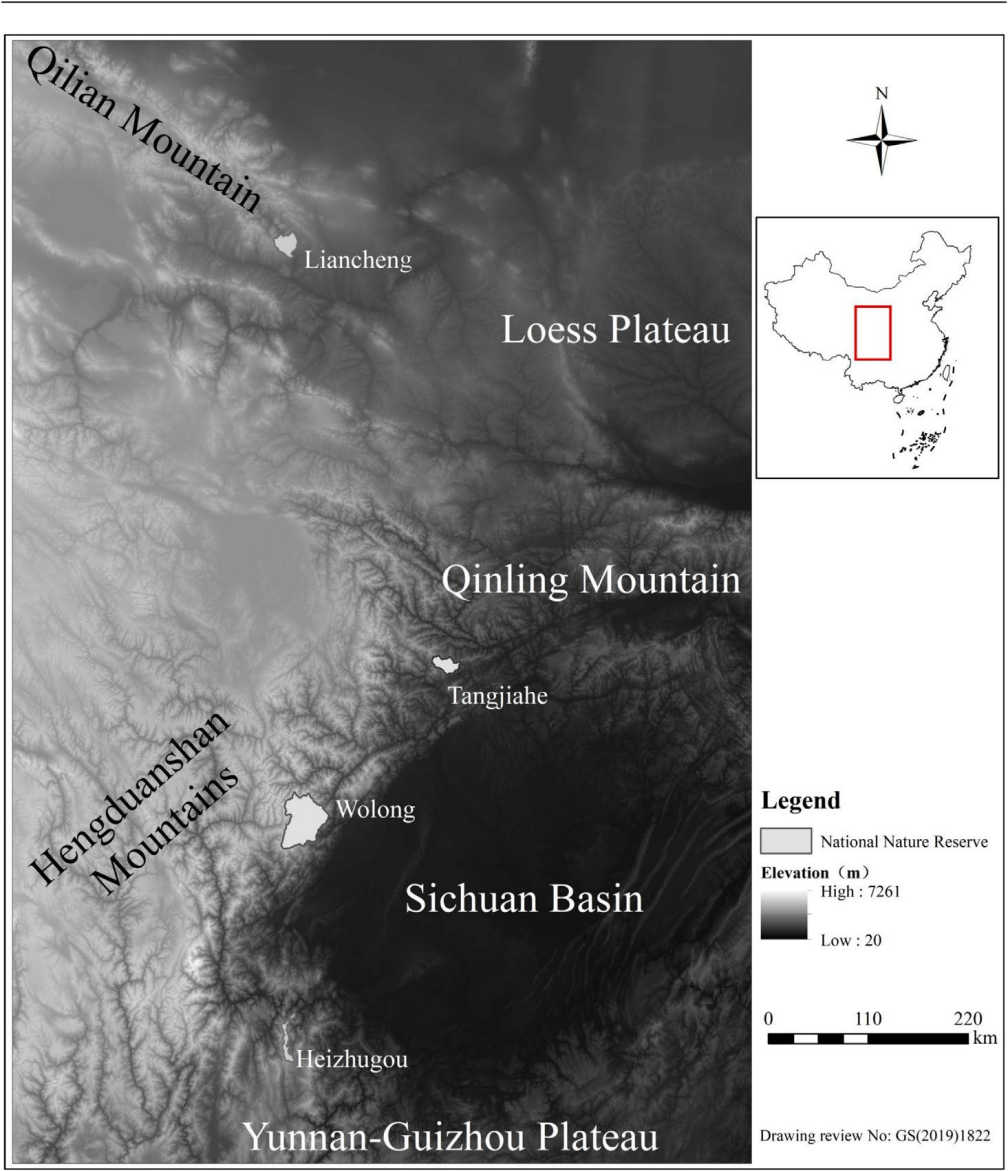
Geographical location of the study area in central China for the study on the spatial association of phasianids from 2017 to 2021.

The study area covers four national nature reserves (NNRs) in central China, surrounding the eastern edge of the Tibetan Plateau, including Liancheng NNR in Gansu province, Tangjiahe, Wolong, and Heizhugou NNRs in Sichuan province (Figure 1). In the north to Qinling Mountains, Liancheng is in the temperate continental climate characterized with cold and dry weather (Z. X. Wang et al. 2024), and located in the transitional zone from the Qilian Mountains southeastward to the Loess Plateau, 102°26′–102°55′ E, 36°33′–36°48′ N, with a size of 479.3 km^2^. The altitude of the NNR ranges from 1870 m above sea level (a.s.l.) in the eastern part to 3616 m a.s.l. in the western part, with a difference of 1746 m (Zhang et al. 2008). Vegetation types include montane forests, steppes, bushes, and meadows vertically distributed along the altitudes (Z. L. Wang et al. 2024). The NNR is in the Palearctic Realm in zoogeography (Li and Dong 2023).

In the south to Qinling Mountains are the Tangjiahe, Wolong, and Heizhugou NNRs. They are located in the Indomalayan Realm in zoogeography (Li and Dong 2023). Tangjiahe, sizing 400 km^2^ in area, is located in the transitional zone from the Qinling Mountains southward to the Sichuan Basin, 104°36′–104°56′ E, 32°30′–32°41′ N. The altitude ranges from 1100 m a.s.l. in the southeast of the NNR to 3864 m a.s.l. in the northwest, with a difference of 2764 m (Wang et al. 2005). It contains diverse vegetation types, changing from evergreen broad‐leaved forests (< 1600 m a.s.l.), evergreen and deciduous broad‐leaved mixed forests (1600–2100 m a.s.l.), coniferous broad‐leaved mixed forests (2100–2400 m a.s.l.), subalpine coniferous forests (2400–3600 m a.s.l.), alpine bushes, and meadows (> 3600 m a.s.l.) (Bai et al. 2021). Wolong, sizing 2000 km^2^ in area, is located in the south to Tangjiahe and in the eastern edge of Hengduanshan Mountains (also called Hengduan Mountains), a mountain area with north–southward huge mountains and valleys in the east to Qinhai‐Tibet Plateau, 102°51′–103°24′ E, 30°45′–31°25′ N, with altitudes ranging 1150 m a.s.l. (southeast)‐6250 m a.s.l. (northwest) and a difference of 5100 m in elevation. The landscape is dominated by high mountains and deep valleys of north–south orientation. The Pitiao River divides the NNR into two parts. The terrain in the northwestern part is steep and largely inaccessible, and the southeastern part is gentler and more accessible (Shi et al. 2017). There are diverse vegetation types ranging from evergreen broad‐leaved forests, evergreen and deciduous broad‐leaved mixed forests, deciduous broad‐leaved forests, coniferous and broad‐leaved mixed forests, and subalpine coniferous forests vertically along the altitudes ending up at 3800 m a.s.l. Further higher are alpine shrubs, meadows, sparse bushes, and screes (Huang et al. 2007). Further south is Heizhugou, an NNR that is 296.43 km^2^ in size and is located on the transitional zone from Yunnan‐Guizhou Plateau northwestward to Hengduanshan Mountains, 102°54′–103°10′ E, 28°39′–29°04′ N. The altitude ranges from 1054 m a.s.l. (southeast) to 4288 m a.s.l. (northwest), with a difference of 2788 m. The landscape is also characterized with mountains and valleys (Lei et al. 2019), but the terrain is gentler and more accessible than that in Wolong. The evergreen broad‐leaved forests, evergreen and deciduous broad‐leaved mixed forests, deciduous broad‐leaved forests, coniferous and broad‐leaved mixed forests, subalpine coniferous forests, shrubs, and meadows are distributed upward along the elevation (Zhu et al. 2019).

The hydrothermal conditions greatly differentiate between the four NNRs. The Qinling Mountains intercept the great amount of warm and humid air currents from the Pacific Ocean. Because of this, compared with that of the other three NNRs, the weather in Liancheng is drier and colder, with an average annual temperature of 7.4°C, an annual evaporation of 1542 mm, and an average annual precipitation of 419 mm (Z. X. Wang et al. 2024). The average annual precipitation in Tangjiahe is 1100 mm, and the annual average temperature is 12°C (Xiao et al. 2024). Although located at lower latitudes, Wolong is in general lower in temperature and humidity than Tangjiahe due to its higher altitudes, with an average annual temperature of only 9.8°C and an average annual precipitation of 931 mm (Zhong et al. 2021). Heizhugou at the southmost is affected by the southeast monsoon, with an average annual temperature of about 14°C and an annual precipitation of 1700–1800 mm (Zhang et al. 2019).

### 2.2 Field Data Collection

We used ArcGIS to generate 1 km × 1 km cells on the maps of the four NNRs, from which we randomly selected ≥ 5% of cells and tried to maximize the number of the cells (i.e., sample size). An infrared camera (model: Yian WS L710 and Ltl Acorn 6210) was installed in each selected cell. Many cells were inaccessible due to terrain restrictions, such as steep cliffs, peaks, and the areas requiring the fieldworkers to hike for ≥ 3 days to reach. When a cell was selected in an inaccessible area, it was abandoned and another cell was randomly selected to replace it if the new cell was located in an accessible place. The distance between two cameras was maintained at ≥ 300 m to minimize the likelihood of two adjacent points occurring in the same small habitat patch, by which to minimize the habitat homogeneity of the data. In Wolong, giant pandas were found in the east to Pitiao River, thus cameras were deployed at this side. Cameras in other NNRs were deployed around all areas. Cameras were installed in the main habitat types of the selected cells and were mounted on tree trunks or other objects 50–80 cm above the ground. The angle of the lens was in parallel to the ground to allow the camera to capture animals on the ground in front scenario and on the shrub canopy in far scenerio, so that image data of terrestrial and semi‐arboreal species could be obtained (Li et al. 2024). Batteries and memory cards were replaced every 3 months. Infrared cameras are triggered by the movement of infrared rays. Ectothermal animals will not trigger infrared cameras. So, only endothermal animals, including birds and mammals, were filmed and present in our database. We used mobile‐built‐in compass to measure coordinates and elevation and the Ovital Map app to measure slope and other habitat dimensions. We estimated vegetation height and canopy coverage by vision.

**FIGURE 2:**
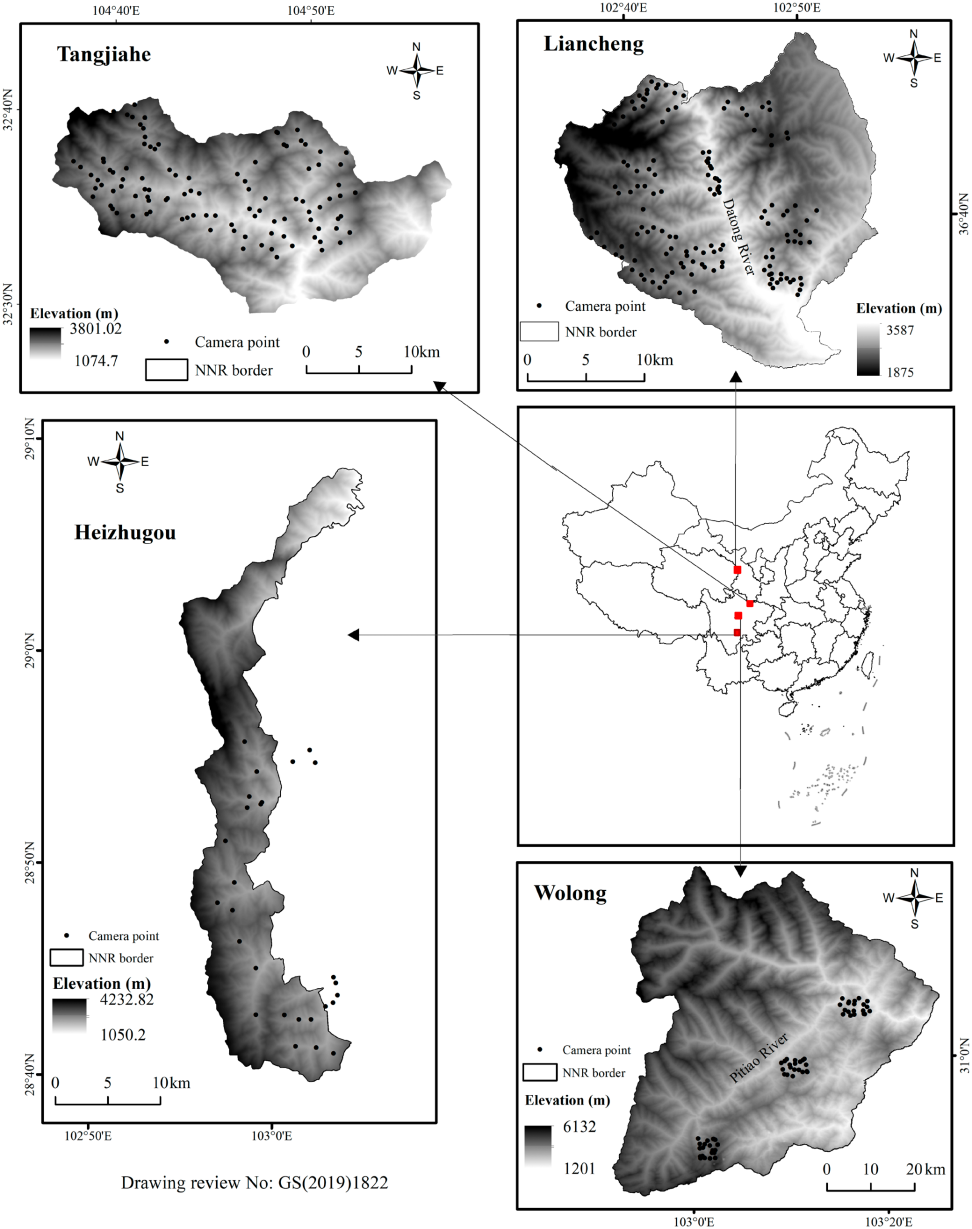
Camera sites for the study on the spatial association of phasianids in Liancheng, Tangjiahe, Wolong, and Heizhugou NNRs in central China from 2017 to 2021 (*n* = 128 in Liancheng, 103 in Tangjiahe, 60 in Wolong, and 28 in Heizhugou).

All cameras worked in the field for ≥ 12 months to obtain complete data on species distribution (Figure 2). They worked at 128 sites in Liancheng from July 2018 to June 2020 (24 months in total), 103 sites in Tangjiahe from September 2019 to December 2020 (16 months), 60 sites in Wolong from February 2017 to April 2018 (14 months), and 28 sites in Heizhugou from September 2019 to December 2020 (15 months). These sites look evenly distributed in general, except for those in Wolong (Figure 2). The uneven distribution pattern in Wolong in Figure 2 was the result of the transformation from an even distribution in 3‐dimensional space to 2‐dimensional map. The cameras were plotted randomly along the mountain surface with huge elevations. Three terraces were recognized at different elevations between which were inaccessible cliffs and valleys. When the camera sites were plotted on the 2‐dimensional map, they appeared in cluster patterns. We have given detailed description on the terrain and the changes in an earlier publication (see Li et al. 2024).

The memory cards were played back on computers for species identification done with reference to Smith and Xie (2009) and MacKinnon and Phillipps (2000). The images did not provide enough morphological details for identifying small murids, so we recorded them as “Muridae”.

Solitary species and social species have different activity patterns, population density, and occurrence frequency. Some images, especially those taken at night, were not good enough in definition for individual identification. Therefore, our data were about the presence or absence of species at each camera site, rather than the occurrence frequency of the species, and thus they were dichotomous data.

Detectability of animals in cameras is always a concern in camera trap techniques for field biological research. Its difference influences the representativeness of species with different detectability in the database which further biases the research results. To minimize the influences, (1) we pointed the camera lenses in parallel to the ground to the rather open space in front of the camera, (2) we set the cameras to work for more than a year to maximize the possibility of a species with less detectability to be present in the database, and (3) we used categorical/dichotomous measures (i.e., present or absent) to rule out the influences on the occurrence frequencies of the species (for details, see Li et al. 2024). Reselection of cells to replace those randomly selected in inaccessible areas may have discounted the randomness of our data, which was a compromise between the statistical requirement and the practical conditions for the use of statistical principles due to the disadvantage of random sampling.

### 2.3 Measuring Interspecific Associations

We used statistical techniques to measure interspecific associations for excluding the co‐occurrences of two species at the same camera sites due to random factors and retaining those due to ecological reasons. According to Siegel and Castellan Jr. (1988) and because of our dichotomous data, we used the Phi coefficient. Before calculating the Phi coefficient for a given pair of species, we counted: (a) the number of camera sites where both species occurred, (b) the number of sites where only one species occurred, (c) the number of sites where only the other species occurred, and (d) the number of sites where both species were absent. We then calculated the Phi coefficient using the counts as follows:

$$r_{ø}=\frac{\mathrm{ad}-\mathrm{bc}}{\sqrt{\left(a+b\right)\left(b+c\right)\left(a+c\right)\left(b+d\right)}}$$

in which *r*_*ø*_ was the Phi coefficient and was used to measure the strength of the association between the two species. The value ranged from 0 (indicating the weakest association) to ±1 (the strongest association). The value of “*ad* − *bc*” > 0 indicated a positive spatial association and that the two species would tend to occur in the same spaces, and the value < 0 indicated a negative association and that the species would tend to occur in the spaces where the other species was absent.

The above calculated Phi coefficient may not necessarily represent the ecological relationship of the species, because different species may co‐occur simply due to random factors, leading to a Phi coefficient. So, it's necessary to do a significance test to eliminate the random factor effects, which was calculated as follows:

$$\chi^{2}=\frac{N{\left(\left|\mathrm{ad}-\mathrm{bc}\right|-0.5N\right)}^{2}}{\left(a+b\right)\left(b+c\right)\left(a+c\right)\left(b+d\right)}$$

where *χ* ^2^ was the chi‐square, and *N* was the total number of camera sites, i.e., *N* = *a* + *b* + *c* + *d*. When *χ* ^2^ < 3.841, the calculated *r*_*ø*_ was rejected, because it was not statistically significant (*p* > 0.05). This *r*_*ø*_ was attributed to random factors. All the *r*_*ø*_ coefficients with *χ* ^2^ ≥ 3.841 (i.e., *p* ≤ 0.05) were retained for assembling species association networks, because they were attributed to ecological requirements.

### 2.4 Assembling Species Association Networks

The retained positive associations were used as edges and the species were used as nodes for assembling networks. We used NetDraw (V2.148) to connect these edges and nodes into spatial association networks.

### 2.5 Analyzing Spatial Association Properties

Species interactions are divided into five categories (Ricklefs and Gary 1999). The first category includes mutualism in which both species benefit from the interaction. In this interaction, the species tend to occur in each other's range and their spatial association shows the property of bidirectional asymmetry. The second category includes commensalism in which only one of the species in the pair in question benefits from the interaction, and the other will neither benefit nor be harmed. In this interaction, the beneficiary species tends to occur in the range of benefiting species and their spatial association shows the property of unidirectional asymmetry. The third category includes predator–prey, herbivore‐plant, and parasite–host interactions in which one species gains benefits and the other is harmed. In such interactions, the beneficiary species tends to occur in the range of the victim species and their spatial association also shows the property of unidirectional asymmetry. The fourth category is the interaction by which both species get harmed, such as competition. If this interaction occurs in a saturated habitat (i.e., there is no empty habitat for either of the species), the species intending to give up the interaction will suffer more than the loss if it maintains the interaction. Therefore, both species tend to persist the interaction and the spatial association shows symmetry. The last category is amensalism in which one species is harmed while the other is not affected. The victim species will avoid spatial overlap with the other and thus there is no spatial association. This interaction is not common in ecological communities. Accordingly, we tested the association properties, so as to explore the possible interaction types that may underly the species pairs. We used the Lambda coefficient to test the properties with our data of dichotomous measures (Siegel and Castellan Jr. 1988), which started with the following contingency table (Table 1).

**TABLE 1:** Contingency table for Lambda coefficient test.

Species *B*	Species *A*		
	*A*_1_	*A*_2_	Total
*B*_1_	*n*_11_	*n*_12_	*R*_1_
*B*_2_	*n*_21_	*n*_22_	*R*_2_
Total	*C*_1_	*C*_2_	*N*

*Note:* *A*_1_ indicated the presence of Species *A* and *A*_2_ the absence of the species. Similarly, *B*_1_ indicated the presence of Species *B* and *B*_2_ the absence of the species. Accordingly, *n*_11_ was the number of camera sites where both species occurred, *n*_12_ and *n*_21_ were the numbers of sites where one species occurred and the other did not, and *n*_22_ was the number of sites where neither species occurred. *R*_*i*_ (*i* = 1,2) and *C*_*j*_ (*j* = 1,2) were the sums of respective columns and rows. *N* was the total number of camera sites.

Based on the table, the Lambda coefficient was calculated as follows:

$$L_{B}=\frac{\sum_{j=1}^{2}n_{\mathrm{Mj}}-\max\left(R_{i}\right)}{N-\max\left(R_{i}\right)}$$

where *n*_*Mj*_ was the largest number in the column j of the contingency table and max (*R*_*i*_) was the maximum sum of rows. *L*_*B*_, the Lambda coefficient, was used to measure the probability that the presence of species A predicts the presence of Species *B*. Accordingly, *L*_*A*_ was coded for measuring the predictability that Species *B* predicts Species *A* and was calculated in a similar procedure after the species for *A* and *B* in the table were switched in the above contingency table. *L*_*B*_ and *L*_*A*_ may not have to be the same.

Similarly, the significance of *L*_*B*_ was tested, starting by calculating the variance of *L*_*B*_ as follows:

$$\mathrm{Var}\;\left(L_{B}\right)=\frac{\left(N-\sum_{j=1}^{2}n_{\mathrm{Mj}}\right)\left(\sum_{j=1}^{2}n_{\mathrm{Mj}}+\max\left(R_{i}\right)-2\sum n_{\mathrm{Mj}}\right)}{{\left[N-\max\left(R_{i}\right)\right]}^{3}}$$

in which, *∑n*_*Mj*_ was the maximum column sum in the row where the maximum *R*_*i*_ was located in the table; i.e., *∑n*_*Mj*_ = *n*_*Mj*_ in this study. This calculation was further followed by:

$$\lambda_{\mathrm{BO}}=L_{B}-z_{p=0.05}\times \sqrt{\mathrm{var}\left(L_{B}\right)}$$

where *λ*_*B*0_ was the predictiveness value of Species *A* to Species *B* with statistical significance, and $z_{p=0.05}$ was the *z* value at *p* = 0.05 (available from the appendix table of Siegel and Castellan Jr. 1988). *L*_*B*_ was significant when *L*_*B*_ > *λ*_*B*0_.

The significance of *L*_*A*_ was tested in a similar procedure.

### 2.6 Phylogeny of Phasianids

We used pair‐wise phylogenetic distance to explore if the phasianids differentiated in space inside communities or in different communities. For this purpose, we downloaded the data of mitochondrial cytochrome b (cytb) gene sequences of the phasianids species found in this study from the National Center for Biotechnology Information (NCBI) database (https://www.ncbi.nlm.nih.gov) to calculate the phylogenetic distances of the species using MrBayes 3.2.7. When two species occurred in the same species network and their phylogenetic distance was shorter than those of the species with other species outside the network, we regarded the two species differentiated inside the same community with evolution to different habitat types. If the distance was greater than those, we regarded their differentiation as the result of overdispersion of the two species coming from different communities and meeting again in evolutionary history.

### 2.7 Statistical Tests

**Chi‐Square Goodness‐of‐Fit Test:** We used the Chi‐square goodness‐of‐fit (Siegel and Castellan Jr. 1988) to test if the observed spatial separations of phasianids were statistically significant. The statistic was calculated as follows:

$$\chi^{2}=\sum_{i=1}^{k}\frac{{\left(O_{i}-E_{i}\right)}^{2}}{E_{i}}$$

where *k* = 2; *O*_*i*_ = the observed number of the *i*th species/genus pairs (*i* = 1 indicated pairs with spatial separation, and *i* = 2 indicated the pairs with spatial association), *E*_*i*_ = the expected number of the *i*th pairs (*E*_*i*_ = the expected number of pairs with spatial separation when *i* = 1, and *E*_*i*_ = the expected number of pairs with spatial association when *i* = 2). According to our research purpose, the null hypothesis H_0_ was that the phasianids were spatially separated due to random factors; i.e., *E*_*1*_ *= E*_*2*_ *= N/2* where *N* = the total number of potential pairs. Accordingly, the alternative hypothesis H_1_ was that they were spatially separated due to evolutionary divergence. When $\chi^{2}\geq 3.841$, *p* ≤ 0.05, H_1_ was accepted, and we regarded the spatial separation as the result due to the evolutionary divergence; otherwise, H_0_ was accepted and we regarded the separation as the result due to random factors.

**Spearman Rank‐Order Correlation Coefficient r_s_:** We used Spearman rank‐order correlation coefficient (Siegel and Castellan Jr. 1988) to test which evolutionary route the Phasianidae takes. The correlation coefficients between the phylogenetic distance and association were calculated for the species with direct associations and the species in different networks. For a given pair of species *X* and *Y*, the phi coefficient between them was directly used in the calculation if they were directly associated. If they were indirectly associated by non‐phasianid species in several ways, we used the strongest association between the two species for the Spearman rank‐order correlation coefficient test. Their association was calculated as follows for each way.

$$r_{ø\left(x-y\right)}=r_{ø\left(x-\mathrm{sp}1\right)}\times r_{ø\left(\mathrm{sp}1-\mathrm{sp}2\right)}\times \ldots \ldots \times r_{ø\left(\mathrm{spi}-y\right)}$$

where *spi* was the *i*th non‐phasianid species between the phasianid species *X* and *Y*. A series of $r_{ø}$ were calculated for each pair of phasianid species and we used the max one for the correlation coefficient *r*_*s*_ tests.

## 3 Results

### 3.1 Phasianid Fauna

**FIGURE 3:**
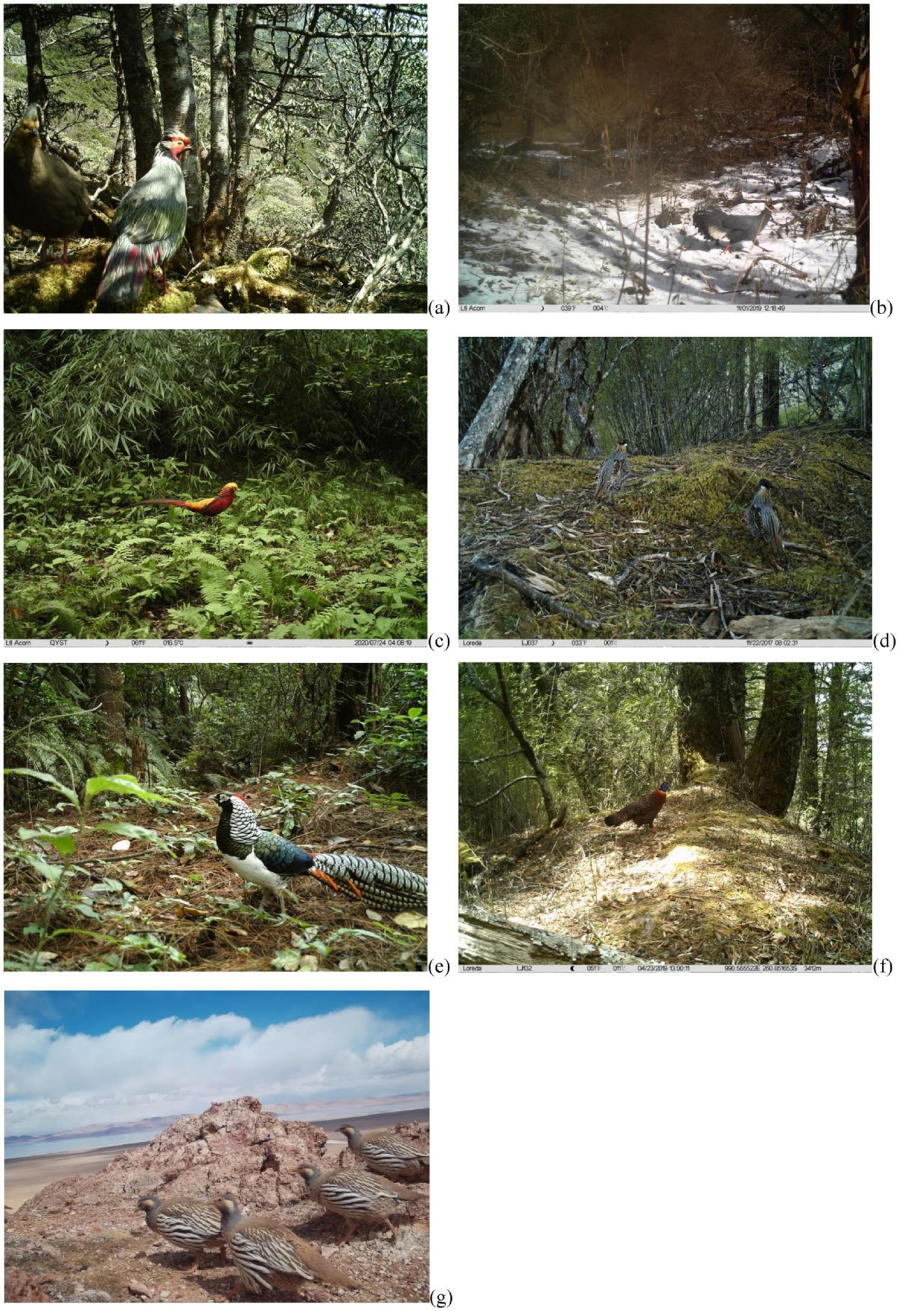
Some phasianids and their habitats in the national nature reserves of Liancheng in Gansu Province, Tangjiahe, Wolong, and Heizhugou in Sichuan Province, China: (a) blood pheasant ( *Ithaginis cruentus* ; BP), (b) blue‐eared pheasant ( *Crossoptilon auritum* ; BEP), (c) golden pheasant ( *Chrysolophus pictus* ; GP), (d) Koklass pheasant ( *Pucrasia macrolopha* ; KP), (e) Lady Amherst's pheasant ( *Chrysolophus amherstiae* ; LAP), (f) Temminck's tragopan ( *Tragopan temminckii* ; TT), and (g) Tibetan snowcock ( *Tetraogallus tibetanus* ; TS) (images from the infrared cameras of this study working in the field in 02/2017–12/2020).

A total of 62 animal species were recorded and identified in the four NNRs, belonging to the avian order Galliformes and five mammalian orders. The avian order contained only one family, i.e., the Phasianidae, with 12 genera and 14 species recorded (Figure 3 for some of the species and their habitats), accounting for about 22% of the 64 phasianid species in China. The phasianids were Temminck's tragopans ( *Tragopan temminckii* ; TT), blood pheasants ( *Ithaginis cruentus* ; BP), ring‐necked pheasants ( *Phasianus colchicus* ; RNP), Chinese monals ( *Lophophorus lhuysii* ; CM), chestnut‐throated partridges ( *Tetraophasis obscurus* ; CTP), Chinese grouses ( *Tetrastes sewerzowi* ; CG), snow partridges ( *Lerwa lerwa* ; SP), Koklass pheasants ( *Pucrasia macrolopha* ; KP), golden pheasants ( *Chrysolophus pictus* ; GP), Lady Amherst's pheasants ( *Chrysolophus amherstiae* ; LAP), white‐eared pheasants ( *Crossoptilon crossoptilon* ; WEP), and blue‐eared pheasants ( *Crossoptilon auritum* ; BEP) from the subfamily Phasianinae, and Przewalski's partridges ( *Alectoris magna* ; PP) and Tibetan snowcocks ( *Tetraogallus tibetanus* ; TS) from the subfamily Coturnicinae (Appendix Table 1).

### 3.2 Spatial Association Networks

The above phasianids occurred in five spatial association networks, including Liancheng, Tangjiahe HAN, Tangjiahe LAN, Wolong HAN, and Wolong LAN. No networks were identified in Heizhugou NNR.

**FIGURE 4:**
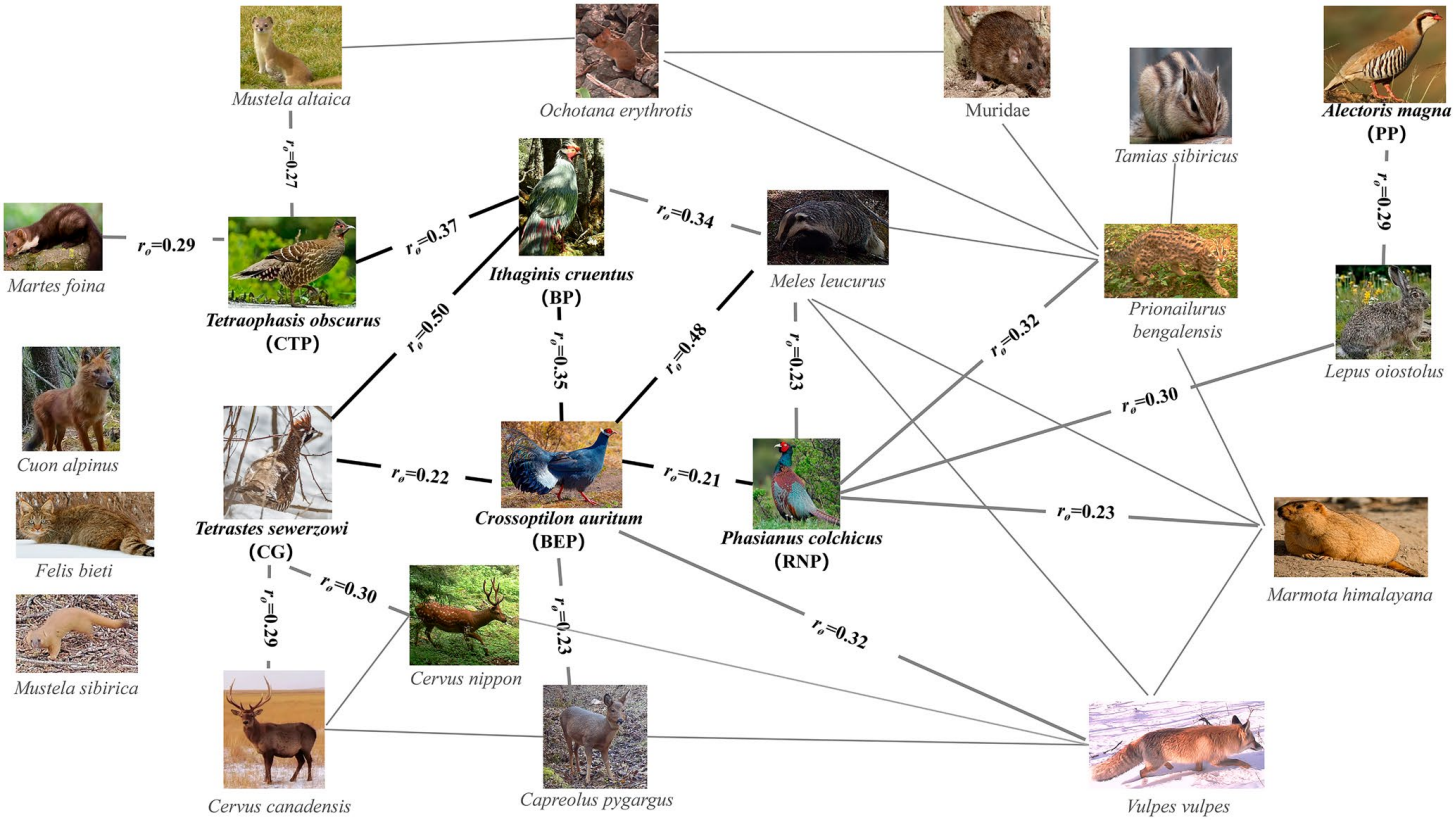
Species associations of phasianids in the Liancheng National Nature Reserve. Each node represents a species recorded within the reserve, with phasianid species shown in bold and non‐phasianid species in gray. Edges connect species pairs whose co‐occurrence was statistically significant according to the phi coefficient (*p* ≤ 0.05). Networks were constructed using presence–absence data from all camera sites operating for ≥ 12 months. The Liancheng network included six phasianid species distributed mainly in deciduous broadleaf forests, coniferous and broadleaf mixed forests, and coniferous forests, between 1977 m and 3126 m a.s.l., together with 16 non‐phasianid species (see text for details). Species codes are provided in Appendix Table 1.

In Liancheng NNR, one spatial association network was identified, which was called Liancheng network and composed of 19 species (Figure 4). The network was distributed at altitudes ranging 1977–3126 m a.s.l., covered mainly with shrubs and coniferous forests. Of the 19 species, six species were phasianids, including five phasianinids (i.e., the BP, BEP, CG, CTP, and RNP), and a coturnicinid (i.e., the PP). The BP (Figure 3a) appeared on deciduous broad‐leaved forests, mixed coniferous and broad‐leaved forests, coniferous forests, deciduous broad‐leaved shrubs and meadows at 2191–3086 m a.s.l. The BEP (Figure 3b) occurred at 2022–3088 m a.s.l. The CG appeared on deciduous broad‐leaved forests, deciduous broad‐leaved shrubs, mixed coniferous and broad‐leaved forests, coniferous forests and meadows at 2270–3086 m a.s.l. The CTP appeared on deciduous broad‐leaved forests, mixed coniferous and broad‐leaved forests, coniferous forests, and meadows at 2323–3088 m a.s.l. The PP appeared on deciduous broad‐leaved shrubs and coniferous forests at 2073–2259 m a.s.l. The RNP appeared on deciduous broad‐leaved shrubs, deciduous broad‐leaved forests, mixed coniferous and broad‐leaved forests, and coniferous forests at 1985–3041 m a.s.l. (Appendix Table 2).

**FIGURE 5:**
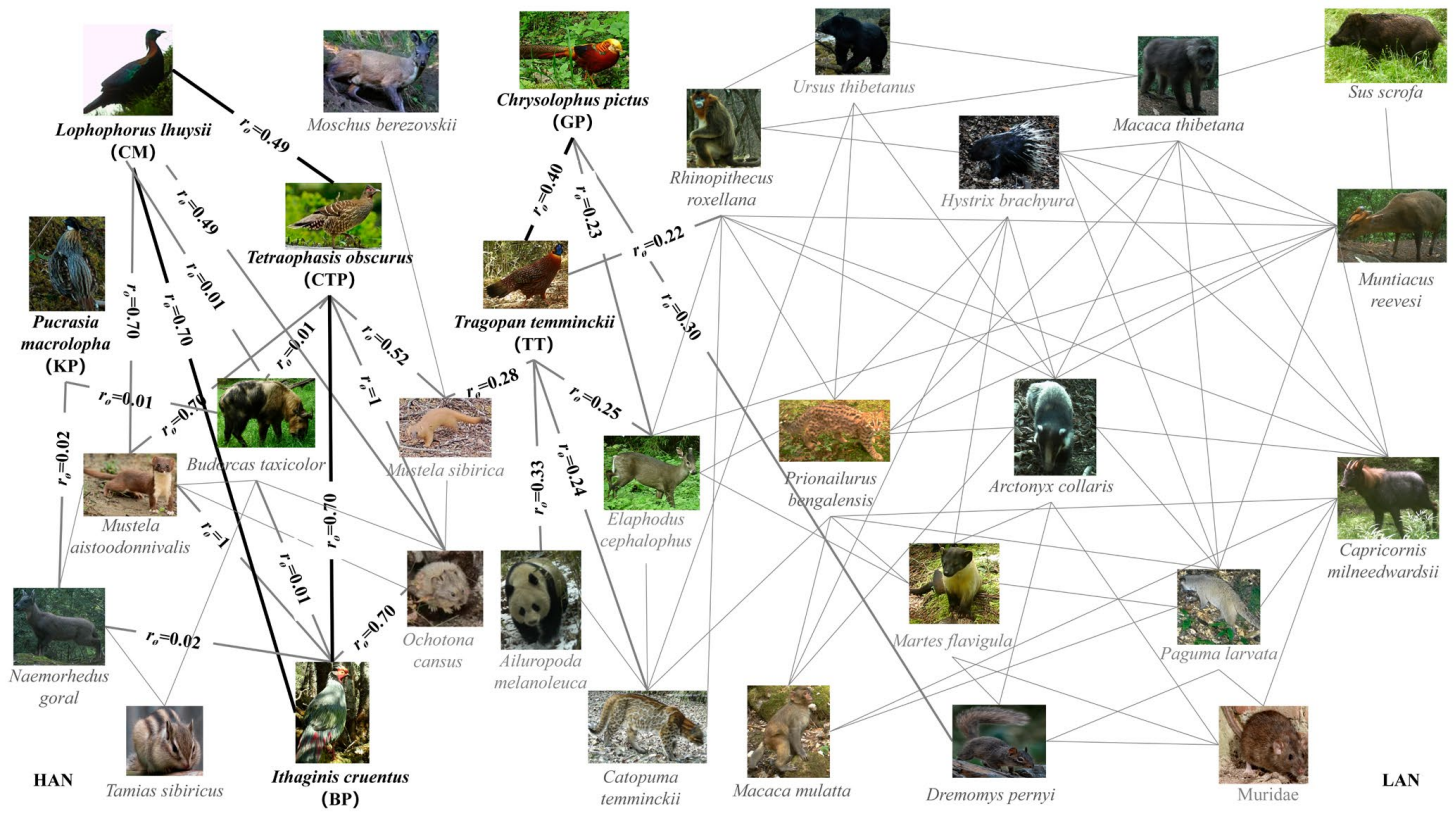
Species associations of phasianids in the Tangjiahe National Nature Reserve. Each node represents a species recorded within the reserve, with phasianid species shown in bold and non‐phasianid species in gray. Arrows indicate asymmetric associations based on *λ* values. Edges connect species pairs whose co‐occurrence was statistically significant according to the phi coefficient (*p* ≤ 0.05). Networks were constructed using presence–absence data from all camera sites operating for ≥ 12 months. The Tangjiahe high‐altitude network (HAN) included four phasianid species distributed mainly in coniferous and broad‐leaved mixed forests between 1497 m and 3373 m a.s.l., together with seven non‐phasianid species, and Tangjiahe low‐altitude network (LAN) included two phasianid species mainly distributed in deciduous broad‐leaved forests and coniferous and broad‐leaved mixed forests between 1541 m and 3041 m a.s.l., and 17 non‐phasianid species (see text for details). Species codes are provided in Appendix Table 1.

In Tangjiahe, a total of 29 species and murids were recorded, including six phasianinids; i.e., the BP, CM, CTP, GP, KP, and TT. Two networks were identified in the NNR, called Tangjiahe HAN and Tangjiahe LAN. The two networks were connected by the association of TT and Siberian weasel ( *Mustela sibirica* ) (Figure 5). Tangjiahe HAN was distributed at 1497–3733 m a.s.l., and the coniferous and broad‐leaved mixed forests were the main habitat type. The network consisted of 11 species, including four phasianinids; i.e., the BP, CM, CTP, and KP. The CM appeared in coniferous and broad‐leaved mixed forests at 3041 m and 3373 m a.s.l. The CTP appeared on 2856–3041 m a.s.l. and BP on 2856 m and 3041 m a.s.l. The KP (Figure 3d) appeared on 1 site at 1497 m a.s.l. (Appendix Table 2). Tangjiahe LAN consisted of 19 species, including two phasianinids, i.e., the TT and GP (Figure 5). The network was distributed at 1220–3041 m a.s.l., with habitat types of deciduous broad‐leaved forests and coniferous and broad‐leaved mixed forests. The TT (Figure 3f) appeared in the vegetation types of coniferous and broad‐leaved mixed forests and deciduous broad‐leaved forests at 1541–3041 m a.s.l. The GP (Figure 3c) appeared in coniferous and broad‐leaved mixed forests and deciduous broad‐leaved forests at 1497–2530 m a.s.l. (Appendix Table 2).

**FIGURE 6:**
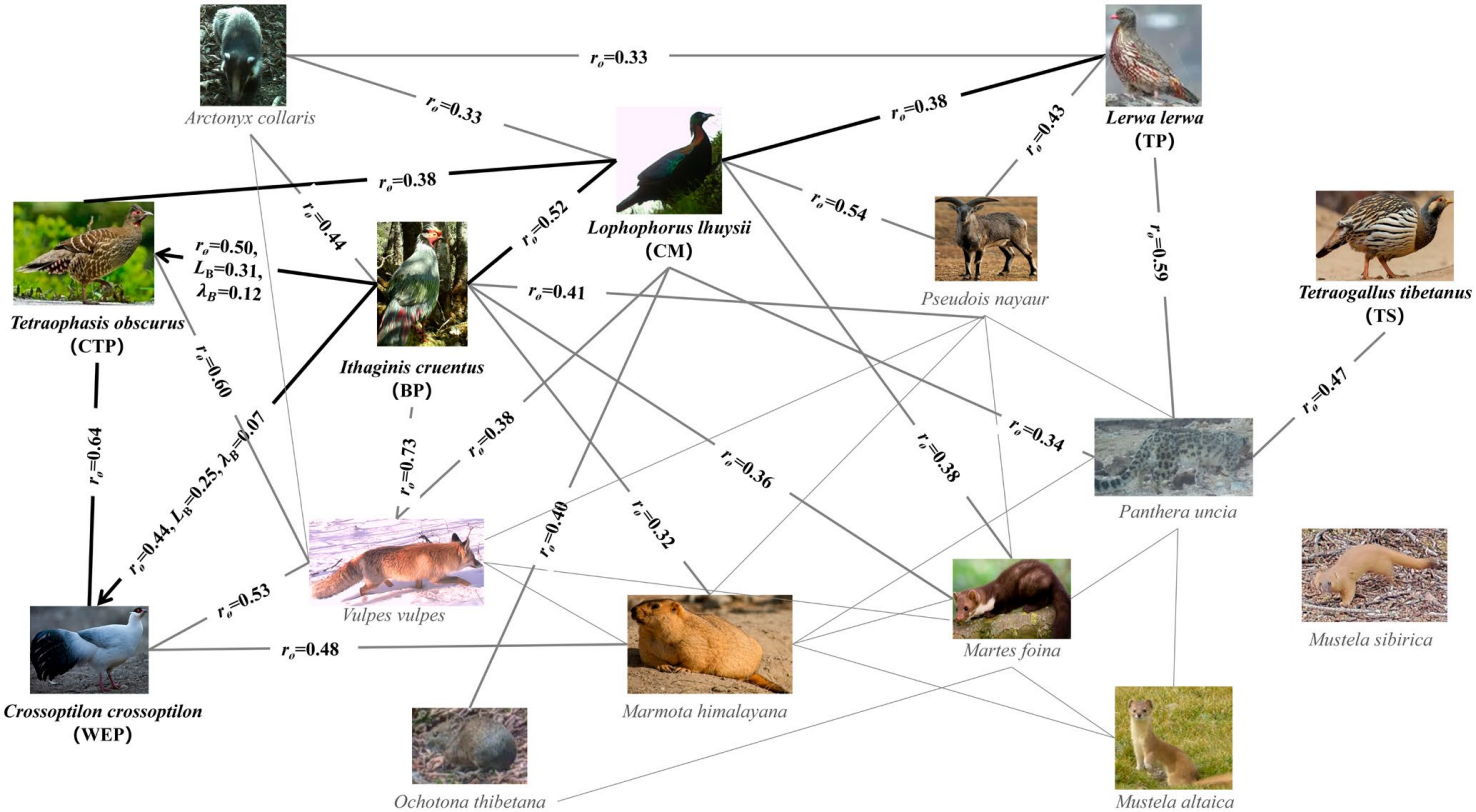
Species associations of phasianids in the Wolong HAN. Each node represents a species recorded within the network, with phasianid species shown in bold and non‐phasianid species in gray. Arrows indicate asymmetric associations based on *λ* values. Edges connect species pairs whose co‐occurrence was statistically significant according to the phi coefficient (*p* ≤ 0.05). The network was constructed using presence–absence data from all camera sites operating for ≥ 12 months. The network included six phasianid species distributed mainly in coniferous and broadleaved mixed forests, coniferous forests, shrub meadows, and screes between 3718 m and 4430 m a.s.l., together with nine non‐phasianid species (see text for details). Species codes are provided in Appendix Table 1.

**FIGURE 7:**
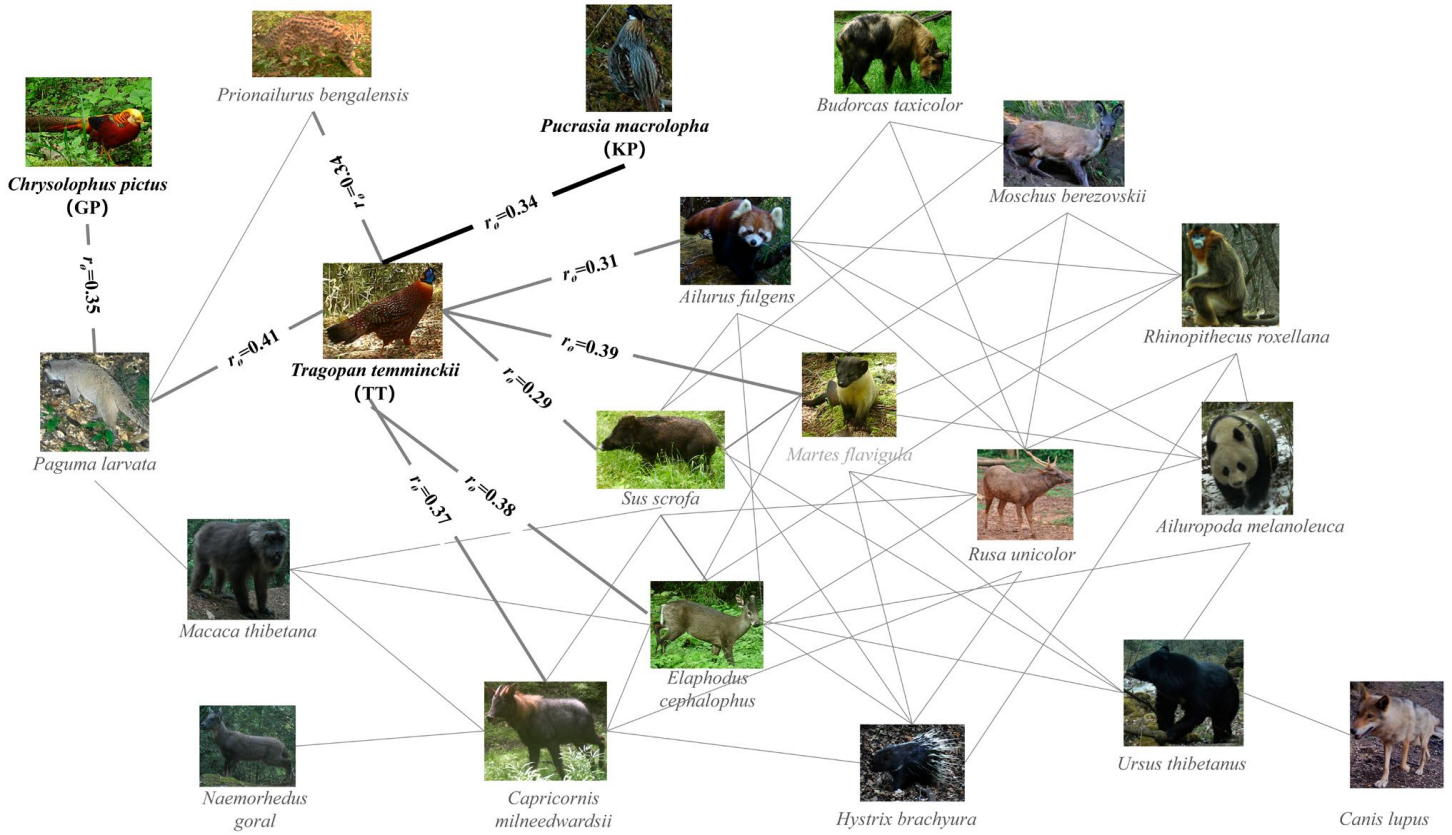
Species associations of phasianids in the Wolong LAN. Each node represents a species recorded in the network, with phasianid species shown in bold and non‐phasianid species in gray. Edges connect species pairs whose co‐occurrence was statistically significant according to the phi coefficient (*p* ≤ 0.05). The network was constructed using presence–absence data from all camera sites operating for ≥ 12 months. The network included three phasianid species distributed mainly in coniferous and broad‐leaved mixed forests between 1749 m and 3361 m a.s.l., together with 17 non‐phasianid species (see text for details). Species codes are provided in Appendix Table 1.

In Wolong, a total of 35 species were identified, including eight phasianinids (i.e., the BP, CM, CTP, GP, KP, SP, TT, and WEP) and a coturnicinid (i.e., the TS). Two networks were identified in the NNR, called Wolong HAN and Wolong LAN (Figures 6 and 7). Wolong HAN was distributed in coniferous and broadleaved mixed forests, coniferous forests, shrub meadows and screes at 3718–4430 m a.s.l., with five phasianinids (i.e., the BP, CM, CTP, SP, and WEP) and the coturnicinid (i.e., the TS) occurring. The BP (Figure 3a) appeared in coniferous and broadleaved mixed forests, coniferous forests, shrub, and meadows at 3042–4303 m a.s.l. The CM appeared in coniferous and broadleaved mixed forests, coniferous forests, meadows, and screes at 3093–4338 m a.s.l. The CTP appeared in shrubs at 3718–4052 m a.s.l. The SP appeared in coniferous forests, shrubs, meadows, and screes at 3186–4430 m a.s.l. The WEP appeared in shrubs at 3718‐3938 m a.s.l. The Coturnicinae TS (Figure 3g) appeared on meadows and screes at 4152 m a.s.l. and 4300 m a.s.l. (Appendix Table 2). Wolong LAN was distributed in habitats dominated by coniferous and broad‐leaved mixed forests at 1749–3361 m a.s.l., with three phasianinids; i.e., the GP, KP, and TT. The GP (Figure 3c) appeared in deciduous broad‐leaved forests, coniferous and broad‐leaved mixed forests, and coniferous forests at 1979–2473 m a.s.l. The KP (Figure 3d) appeared in deciduous broad‐leaved forests, coniferous and broad‐leaved mixed forests, and shrubs at 2272–3718 m a.s.l. The TT (Figure 3f) appeared in evergreen broad‐leaved forests, deciduous broad‐leaved forests, coniferous and broad‐leaved mixed forests, and coniferous forests to shrubs at 1802–3718 m a.s.l. (Appendix Table 2).

In Heizhugou, a total of 28 species were recorded, but no networks were identified. Three phasianinids were recorded in the NNR, including the BP, LAP, and TT. The LAP (Figure 3e) appeared in evergreen and deciduous broad‐leaved mixed forests, coniferous and broad‐leaved mixed forests, coniferous forests, and bamboo forests at 2260–2881 m a.s.l. The TT (Figure 3f) appeared in deciduous broad‐leaved forests, coniferous and broad‐leaved mixed forests, coniferous forests, and bamboo forests at 2263–3018 m a.s.l., and the BP appeared in coniferous and broad‐leaved mixed forests, coniferous forests, and bamboo forests at the same altitudes. According to the altitudinal range of Heizhugou (1054–4288 m) and vegetation types, all the phasianids were distributed in the middle altitudinal section, but the LAP tended to occur in warmer and lower altitudes, whereas the TT and BP (Figure 3a) tended to occur in colder and higher altitudes (Appendix Table 2).

### 3.3 Spatial Relationship Between Phasianids

A total of 14 phasianids species were identified in our study area which resulted in 91 potential species pairs (i.e., *N* = 91). There were 12 spatial associations that were actually observed (i.e., *O*_2_ = 12) between the species BP, BEP, CG, CM, CTP, GP, KP, SP, TT, and WEP, all from the subfamily Phasianinae. Some of the associated species pairs occurred in more than one network. There were accordingly 79 species pairs with spatial separation (i.e., *O*_1_ = 79). Thus, *E*_1_ = *E*_2_ = 91/2 = 45.5. The chi‐square goodness‐of‐fit test indicated that the spatial separation between phasianids could not be attributed to random factors ($\chi^{2}=49.329,p\leq 0.01$); instead, the phasianids strongly and significantly tended to avoid spatial association from each other.

The 12 species associations actually represented 11 genus pairs between 10 genera, including *Crossoptilon*, *Crysolophus*, *Ithaginis*, *Lerwa*, *Lophophorus*, *Phasianus*, *Pucrasia*, *Tetraophasis*, *Tetrastes*, and *Tragopan*. The 10 genera made up 45 potential geneus pairs (*N* = 45). The genus pairs (*n* = 11) accounted for 24.4% of the number of potential pairs. In the chi‐sqare goodness‐of‐fit test, *E*_1_ = *E*_2_ = 45/2 = 22.5, *O*_1_ = 34, *O*_2_ = 11. The test result similarly indicated a strong and significant tendency the phasianids avoided spatial association from each other ($\chi^{2}=11.756,p\leq 0.01$).

In Liancheng, five spatial associations were found between the phasianinids (Appendix Table 3), including the species pairs of (1) the BP and CTP, (2) the BP and CG, (3) the BP and BEP, (4) the BEP and CG, and (5) the BEP and RNP. All the associations were symmetrical. The spatial separation existed between the Coturnicinae PP and any other phasianids. The BP co‐occurred with the CTP, CG, and BEP in coniferous and broadleaf mixed forests, coniferous forests, deciduous broadleaf forests, and meadows, but the main habitat types varied between different pairs. Those of the BP with CTP occurred mainly in the coniferous and broadleaf mixed forests, coniferous forests, and deciduous broadleaf forests, and with CG and BEP mainly in the coniferous forests and deciduous broadleaf forests. The BEP co‐occurred with CG and RNP in bush, coniferous and broadleaf mixed forests, coniferous forests, deciduous broadleaf forests, and meadows respectively at 2270–2928 m a.s.l. and 2142–3026 m a.s.l., but the main habitat types were bush and coniferous forests (Appendix Table 4).

In Tangjiahe HAN, three associations were found between the phasianinids, including the species pairs of (1) the BP and CTP, (2) the BP and CM, and (3) the CTP and CM (Appendix Table 3). The BP and CTP were asymmetrically associated, in which the CTP tended to occur in BP's range. The associations of the CM with BP and CTP were symmetric. In Tangjiahe LAN, the GP and TT were symmetrically associated. The KP was spatially separated from all other phasianids. The BP and the CTP co‐occurred at 2856–3041 m a.s.l., and the CTP and CM at 2894–3041 m a.s.l. The GP and TT co‐occurred in coniferous and broadleaf mixed forests and deciduous broadleaf forests at 1570–2202 m a.s.l. (Appendix Table 4).

In Wolong HAN, six spatial associations were found between the phasianinids, including the species pairs of (1) the BP and CTP, (2) the BP and CM, (3) the BP and white‐eared pheasant (WEP), (4) the CTP and CM, (5) the CTP and WEP, and (6) the CM and SP (Appendix Table 3). Here, the BP and CTP were asymmetrically associated, and the BP tended to occur in the range of CTP, which was reverse to the association in Tangjiahe HAN. The BP and WEP were also asymmetrically associated, in which BP tended to occur in WEP's range. In Wolong LAN, the KP was symmetrically associated with the TT (Appendix Table 3). The TS was spatially separated from all phasianinids in Wolong HAN, and there was no association between the GP and other phisianids in Wolong LAN. The BP and the CTP co‐occurred in bush at 3718–4052 m a.s.l., the BP and CM in bush, coniferous and broadleaf mixed forests, coniferous forests, meadows, and screes at 3186–4300 m a.s.l., with the major habitat type of bush. The BP and WEP co‐occurred in bush at 3718–3938 m a.s.l., and the CTP and CM in the same type but at 3718–4052 m a.s.l. The CTP and WEP co‐occurred in bush at 3718–3938 m a.s.l., and the CM and SP in bush, coniferous forests, meadows, and screes at 3186–4300 m a.s.l. The KP and TT co‐occurred in coniferous and broadleaf mixed forests at 2272–3718 m a.s.l. (Appendix Table 4).

**FIGURE 8:**
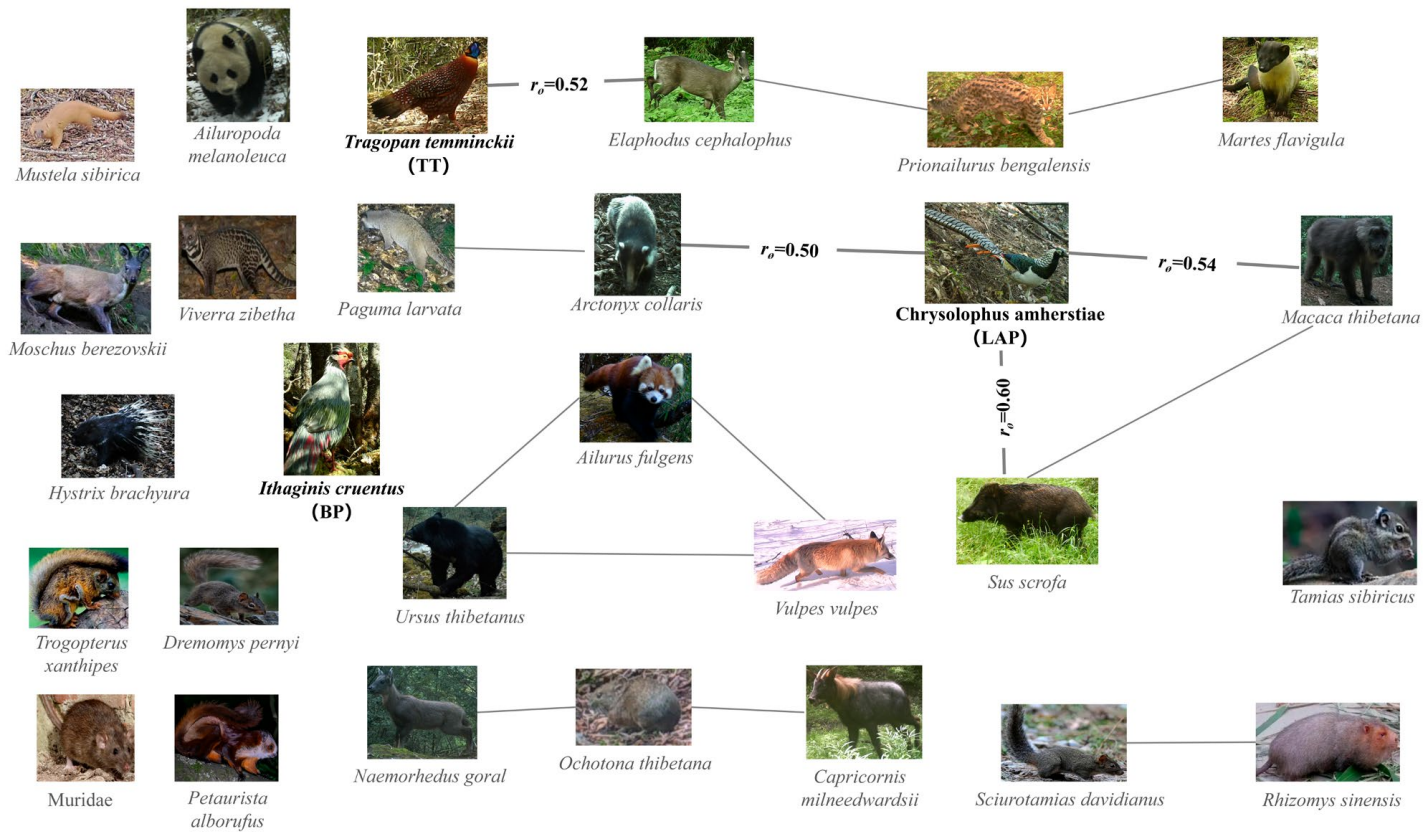
Species associations of phasianids in the Heizhugou NNR. Each node represents a species recorded in the network, with phasianid species shown in bold and non‐phasianid species in gray. Edges connect species pairs whose co‐occurrence was statistically significant according to the phi coefficient (*p* ≤ 0.05). All cameras were operating for ≥ 12 months to collect presence‐absence data. A total of three phasianid species were recorded in evergreen and deciduous broad‐leaved mixed forests, deciduous broad‐leaved forests, coniferous and broad‐leaved mixed forests, coniferous forests, and bamboo forests between 2260 m and 3018 m a.s.l. together with 25 non‐phasianid species (see text for details). Species codes are provided in Appendix Table 1.

No networks were identified in Heizhugou NNR, and the three phasianids were not directly associated spatially (Figure 8).

### 3.4 Phylogenetic Distances of Phasianids

The results of phylogenetic distance showed that the distances between species (i.e., the GP‐LAP and BEP‐WEP) was below 0.1, with a mean of 0.03 (Appendix Table 5). Those between genera ranged from 0.14 (e.g., those between the WEP and the GP and LAP, and between the RNP and the GP and LAP) to 0.48 (i.e., the distance between the BP and CG), with a mean of 0.32. The distances between different subfamilies ranged from 0.42 (i.e., those between the PP and the SP and CM) to 0.55 (i.e., the distance between the TS and CG), with a mean of 0.49. The differences followed the general tendency of evolutionary divergence. The 12 direct spatial associations occurred only between the species across different genera of the Subfamily Phasianinae, with distances between the species of the pairs ranging from 0.16 (the distance between the RNP and BEP) to 0.48 (between the BP and CG) and a mean of 0.35. The result of Spearman rank‐order correlation coefficient test showed that their phylogenetic distance was not correlated with the strength of their associations (*r*_*s*_ = 0.3986, *n* = 12, one‐tailed, *p* > 0.05). However, the correlation varied with different networks. In Liancheng, the correlation coefficient was significant (*r*_*s*_ = −0.4518, *n* = 15, one‐tailed, *p* < 0.05), indicating that the phylogenetic distance was negatively correlated with the spatial association of the phasianids in the network. Tangjiahe HAN and Tangjiahe LAN were connected by the association of TT and Siberian weasel ( *Mustela sibirica* ), so the phasianids in the two networks were lumped together for their association calculation. The result showed no significant correlation between the distance and the association of the phasianids in Tangjiahe NNR (*r*_*s*_ = 0.0580, *n* = 15, one‐tailed, *p* > 0.05). Similar relationship also existed in the phasianids in Wolong HAN (*r*_*s*_ = −0.3643, *n* = 15, one‐tailed, *p* > 0.05). There were too few phasianid pairs in Wolong LAN (*n* = 3, < 5), so no correlation coefficient was calculated for the network.

## 4 Discussion

Different population densities and activity patterns may influence the detectability of different species. Imperfect detections may influence co‐occurrence estimates, and thus further influence the final results. Therefore, we set the cameras to work for more than a year and used categorical measures to minimize the influences. The reason for doing so is that the activity patterns and population densities change with seasons and the year‐cycle will cover the time that a species appears in a given camera with greatest likelihood. Categorical measures do not count the occurring frequencies, which further minimizes the influences. According to Chen et al. (2017), Wang et al. (2019), Xiao et al. (2025), Li (2023), Wei et al. (2021), Shi et al. (2017), and Zhang et al. (2020), there are 4 other phasianid species found in the NNRs, including Sichuan partridges ( *Arborophila rufipectus* ) and silver pheasants ( *Lophura nycthemera* ) in Heizhugou NNR, Tibetan partridges ( *Perdix hodgsoniae* ) in Wolong NNR, and Chinese bamboo partridges ( *Bambusicola thoracica* ) in Heizhugou and Wolong NNRs, in addition to the 14 species reported in the results of this paper. The Sichuan partridge is found outside Heizhugou NNR (Zhang et al. 2020), whereas our infrared cameras were all working inside the NNR. The silver pheasant is distributed in extremely low density in the fringes of the NNR where natural vegetation and artificial plantation meet (Zhang et al. 2020). The Tibetan partridge and the Chinese bamboo partridge are reported as new records of the species (i.e., the species are not reported in earlier surveys) in Wolong NNR (Li 2023; Zhang et al. 2020; Wei et al. 2021), indicating rare visits of the species to the NNR. In Heizhugou NNR, the Chinese bamboo partridge is found in the marginal habitats at 800–1200 m a.s.l., including the cliff of gorges and roadside bushes outside the NNR (Zhang et al. 2020) where we had no cameras installed due to the difficulty of access and human disturbance. Rare visits (camera traps of 1 or 2) have also been reported for the BEP (see Liancheng Network, Figure 3) and the TS (see Wolong HAN, Figure 5) in Tangjiahe (Xiao et al. 2025; Tangjiahe NNR website, January 26, 2024), but the species did not appear in Tangjiahe networks. Therefore, the species native to the NNRs have all appeared in our data sets, and the species occasionally visiting the NNRs did not appear, indicating that they may not influence the spatial association patterns of the local communities.

Based on our research findings, our study has tested the prediction that the extant phasianids extensively occurred in different geographical spaces with spatial separation between them, although a few associations were found between the species. The separation may originate in evolutionary divergence. Differences in ecological preference may alleviate competition between the phasianids with spatial associations.

### 4.1 Evolutionary Divergence of Phasianids

#### 4.1.1 Spatial Separation Between Species

Sibling species evolve directly from the divergence of their ancestral species population. Thus, they have experienced short time of divergence. Most extant phasianids diverged in Pleistocene (Li and Dong 2023). The ring‐nected pheasant *Phasianus colchicus* (RNP), for example, diverged in around 1.6 Ma (Kayvanfar et al. 2017), the golden pheasant *Chrysolophus pictus* (GP) and the Lady Amherst's pheasant *Chrysolophus amherstiae* (LAP) in 1.75 ± 0.40 Ma (Huang et al. 2006), the snow partridge *Lerwa lerwa* (SP) in 0.44–0.4 Ma (Yao 2017), and the blue‐eared pheasant *Crossoptilon auritum* (BEP) in 0.3 Ma (Li et al. 2015). The short time of divergence is indicated in their phylogenetic distance. In this study, the phylogenetic distance between GP and LAP was 0.01, the shortest one among the 91 potential species pairs; and the distance between the white‐eared pheasant *Crossoptilon crossoptilon* (WEP) and BEP was 0.05, similar to that of GP‐LAP. Therefore, the sibling species share most features in morphology, physiology, and ecological requirements, and will be faced with severe completition when encountering, which is detrimental to the diversity of their genus (competitive exclusion; Gause 1936; Ricklefs and Gary 1999; Schluter 2000; Rundle and Nosil 2005; Futuyma 2013; Heams et al. 2017). The best evolutionary strategy for the genus is that the species evolve adaptations to different ecological communities, and thus only one species of the genus will appear in a community. In this study, we recorded 12 phasianid genera, with 10 genera each containing only one species, accounting for 83% of the total number of genera. Only *Chrysolophus* and *Crossoptilon* contained two species each; i.e., the GP and LAP of *Chrysolophus* and WEP and BEP of *Crossoptilon*. The BEP diverges in 1.2–1.6 Ma (Wu et al. 2005) and then disperses northward and evolves adaptation to the dry‐cold habitats in the Central Asian of the Palearctic Realm (Lu et al. 1998; Zheng 2015). The WEP diverges from *Crossoptilon harmani* (the earliest species of the genus; Hebert et al. 2004) in Hengduan Mountains around 0.246 Ma (Wang et al. 2018), and disperses and evolves adaptation to the humid‐cold habitats in Hengduan Mountains (Lu et al. 1998). The WEP and BEP are currently distributed in different ecological communities, which may be driven by their adaptation to different humidity. The GP and LAP diverged in the early Pleistocene, approximately 1.75 ± 0.40 Ma (Huang et al. 2006) in the south to Qinling Mts and in Hengduan Mountains due to glacial isolation (Xiang et al. 2000). The GP is currently distributed in a variety of mountain habitat types in the south to Qinling Mts and the eastern monsoon region of China, indicating its ancestral adaptation to warm and humid climates. It is also found in the north to Qinling, which may be the result of the southern fauna dispersing northward in Holocene (Li and Dong 2023). The LAP is also distributed in the warm and humid habitats in southwestern China (Zheng 2015). The two species appeared in different networks in our study, indicating their vicarious distribution pattern.

As a consequence, all genera in any of our networks contained only one species each, although eight of the 12 genera each contain more than one species in taxonomy. This strategy may have effectively made the species avoid severe competition from the same genus. Such monotypicalness has also been found in our study on mustelid mammals (Liu et al. 2024).

#### 4.1.2 Spatial Separation Between Higher Taxa

In further divergence, the sibling species will accumulate more differences in morphological, physiological, and ecological features, which makes them to be more and more different at genus and further higher levels. Animals faced with higher ecological pressures evolve faster than those with lower pressures (Li and Dong 2023); thus, different phasianid taxa have experienced different time of evolution, and their positions in the phylogenetic tree determines their phylogenetic distance (Sangster et al. 2023). Our results showed the distance ranged from 0.14 to 0.48 for genera, overlapping with part of the range of subfamilies that ranges from 0.42 to 0.55. It has been suggested that the family Phasianidae originated and differentiated in the Southeast Asia in Paleogene (Mayr and Weidig 2004; Zheng 2015) and dispersed across Eurasia to Africa, Australasia, and North America (Wang et al. 2013). The subfamilies Phasianinae and Coturnicinae had existed in some 28.1 Ma (Chen et al. 2021). The oldest fossils of Asian Coturnicinae were found in the Burdigalian deposit of early Miocene (20.44–15.97 Ma) in Jiangsu, and the oldest fossils of Phasianinae were found in the Tortonian deposit of late Miocene (11.62–7.426 Ma) in Yunnan, China (https://Paledbiodb.org). This indicates that the spatial separation between the two subfamilies originate in their evolutionary divergence. Jiangsu experienced temperature declining after the Burdigalian and it was becoming cold and arid; whereas it was warm and humid in Yunnan in the same time (Li and Dong 2023). Thus, coturnicinids may have experienced evolution in cold climates, and phasianinids in warm climates. Our results showed that coturnicinids (i.e., the Przewalski partridge *Alectoris magna* ‐PP and the Tibetan snowcock *Tetraogallus tibetanus* ‐TS) occurred in cold communities (i.e., Liancheng and Wolong HAN). With the global cooling in Pliocene, climate started to differentiate between the two sides of Qinling Mts., with the cold and dry weathers in the north and warm and humid weathers in the south, which drove the fauna to differentiate into Palearctic in the north and Indomalayan in the south. The high altitudes of the Hengduan Mountains starting to rise in Oligocene formed habitat corridors for Palearctic fauna, probably including the ancestor of *Tetraogallus*, to disperse southward since Pliocene (Li and Dong 2023). It is suggested that the *Tetraogallus* originates locally in Hengduan Mountains in Pleistocene (Ding et al. 2020), and the *Alectoris* in the Pleistocene Mediterranean basin (Kabasakal et al. 2025). Our results showed that PP and TS lived in similar habitat types (i.e., similar ecological requirements) but in different geographic spaces (i.e., geographical separation), by which to avoid competition. We suggest that the spatial separation between them comes from the evolutionary divergence of their ancestors.

The weather maintained warm since Pliocene in Yunnan, including the low altitudes of the Hengduan Mountains part in NW Yunnan. The Phasianinae continued to diversify. Some of the offsprings evolved adaptation to cold environment and dispersed northward and vertically to high altitudes of the Hengduan Mountains to join Palearctic fauna. For example, the genus *Lophophorus* appeared in 3 Ma (Zhan and Zhang 2005; Cui et al. 2019). A molecular phylogenetic study suggests that the genus might originate locally in the Hengduan mountains (Zhan et al. 2003). Our results showed that one of its members (i.e., the Chinese monal *L. lhuysii* ‐CM) shared the same network with the TS in Wolong HAN, but was not directly associated in space. The CM appeared in a range of habitat types, but mainly in forests; whereas the TS appeared in open spaces of meadows and screes (Zheng 2015). Another example is the genus *Crossoptilon* which appeared in 1.6–1.2 Ma (Wu et al. 2005). A molecular phylogenetic study suggests that the genus might originate in the boundary region of Sichuan, Yunnan, and Tibet (Wu et al. 2005). One of its members, the blue‐eared pheasant *C. auritum* ‐BEP, shared the same network with the PP in Liancheng, but was not spatially associated with it. The distribution of BEP biased in high altitudes, whereas that of PP in low altitudes (Zheng 2015). Other phasianinid genera appearing in Liancheng and Wolong HAN included *Ithaginis*, *Tetraophasis*, *Tragopan*, *Tetrastes*, *Phasianus*, and *Lerwa*. They originate in different areas of Palearctic Realm in different evolutionary time (Randi et al. 2000; Potapov 2002; Kayvanfar et al. 2017; Song et al. 2021; also see the data base of Mindat.org at https://www.mindat.org/taxon‐2473738.html). They were not spatially associated with the coturnicinids due to similar differences in habitat requirements (Appendix Table 3).

Other phasianinids retain the adaptation to warm and humid weathers, a primitive ecological feature evolved in the long time of Neogene. These genera join Indomalayan fauna, including *Pucrasia* and *Chrysolophus* recorded in this study. It seems that *Tragopan* and *Ithaginis* have adaptations to a wider temperature zone, because we have also recorded them from the warmer environment of Wolong LAN, Tangjiahe LAN, and Heizhugou NNR. It is suggested that the genera diverge in different times and different areas around the Himalayas (Huang et al. 2006; Huang and Ke 2013). The *Chrysolophus* and *Pucrasia* in Wolong LAN appeared in similar habitat types, but the *Chrysolophus* was mainly found in forests at lower altitudes; whereas the *Pucrasia* appeared in shrubs at higher altitudes in addition to the forests below. So, it is inferred that the differentiated habitat preferences make the genera avoid severe competition and thus be able to co‐occur in the same community after meeting again in evolutionary history.

### 4.2 Evolution of Spatial Associations

Spatial separation keeps sibling species (e.g., the WEP and BEP of *Crossoptilon* or GP and LAP of *Chrysolophus* in this study) away from severe competition with each other. With further evolution, the genera will be faced with a choice of either further keeping space divergence or co‐occuring in geographic space but with differences in ecological niche dimensions. In this study, the Spearman rank‐order correlation coefficient test showed no relationship between the phylogenetic distance and spatial association of the 12 associated species pairs, indicating a general tendency of diversity development by ecological niche differentiations (Route 2). However, a significant correlation was found in Liancheng network (Route 1), which was different from that in the networks in Tangjiahe and Wolong where no correlation was found (Route 2). Liancheng is located in the Palearctic realm, and Tangjiahe and Wolong are located in Indomalayan realm. The periodic glaciers in Pleistocene repeatedly wipe out the Palearctic biota, which leaves large amount of empty space for the Indomalayan biota to disperse northward in the Holocene; whereas the Indomalayan biota continuously evolve (Li and Dong 2023). Because of the difference in evolutionary history, the empty space in Palearctic may allow the phasianid genera to further diverge in space to further develop biodiversity. In the space‐saturated Indomalayan, the divergence may not be allowed; thus, the genera may evolve ecological differentiations to co‐exist to further develop biodiversity. The 12 species associations represented 11 genus pairs. Of the genus pairs, two were asymmetric associations from which one of the genera of the pair may benefit from their interactions. Nine associations were symmetric in which the genera may obtain different resources from the space they co‐exist.

#### 4.2.1 Assymetric Associations of *Tetraophasis*‐*Ithaginis* and *Ithaginis*–*Crossoptilon*

The association of *Tetraophasis*‐*Ithaginis* occurred in Liancheng Network, Tangjiahe HAN, and Wolong HAN, and the *Ithaginis*‐*Lophophorus* occurred in Tangjiahe HAN and Wolong HAN, demonstrating the companionship of these genera at various extents. Molecular phylogenetic studies suggest that *Ithaginis* and *Lophophorus* are sibling groups (Bush and Strobeck 2003; Kimball and Braun 2008), and *Tetraophasis* and *Lophophorus* belong to the same evolutionary clade (Shen et al. 2010; Wang et al. 2013), implying that the three genera may not have fully differentiated in spatial distribution. The unidirectional asymmetric association between *Ithaginis* (BP in this association) and *Tetraophasis* (CTP in this association) differed with communities. In Tangjiahe HAN (Figure 5), *Ithaginis* attracted *Tetraophasis*; whereas in Wolong HAN (Figure 6), *Tetraophasis* attracted *Ithaginis*. Our earlier analyses suggested that the species attracting may benefit to the species attracted and facilitate the latter to survive (Wang et al. 2023; Li et al. 2024; Liu et al. 2024). This implies that the *Ithaginis‐Tetraophasis* association may be beneficial to the diversity of Phasianidae. Similarly, the unidirectional asymmetric *Ithaginis*‐*Crossoptilon* may be also beneficial to the diversity. In analyzing the spatial relationship of mustelid mammals, we found asymmetrical associations in the genus pairs of *Martes* ( *M. foina* , the stone marten)—*Mustela* ( *M. altaica* , the Altai weasel) in Wolong HAN and *Martes* ( *M. flavigula* , the yellow‐throated marten)—*Arctonyx* ( *A. collaris* , the hog badger) in Tangjiahe LAN (Liu et al. 2024). The hog badger tended to occur in the range of the yellow‐throated marten perhaps due to the benefits from the seed dispersing by the marten. The stone marten tended to occur in the weasel's range, because the marten kills the weasel to eliminate competition. However, the pair of *Martes*‐*Mustela* (*n* = 1) accounts for only a small proportion of the potential pairs between mustelids (*n* = 21), and thus may not significantly impact the biodiversity of Mustelidae.

#### 4.2.2 Symmetric Associations

The other nine genus pairs may indicate severe competition. However, genera evolve in longer time than species, which allows them to evolve more differentiations in other niche dimensions to alleviate competition, so that they become able to coexist. In Wolong HAN, for example, the *Lophophorus* (i.e., the CM) was spatially associated with the *Lerwa* (the SP). However, ecological studies show that the SP primarily feeds on mosses, lichens, and berries; whereas the CM consumes woody plants (Zheng 1987; Lu 1991), indicating that the dietary differentiation supports the two genera to co‐occur. The dietary differentiation also exists between the genera of the pairs of *Lophophorus* (CM)‐*Ithaginis* (BP), *Lophophorus* (CM)‐*Tetraophasis* (CTP), *Crosoptilon* (WEP)‐*Tetraophasis* (CTP), and *Crosoptilon* (WEP)‐*Ithaginis* (BP) (Zheng 1987, 2015; Lu 1991).

The *Phasianus* (i.e., RNP) is widely distributed in diverse range of habitats (Peng et al. 2025). All the habitats are characterized with dense vegetation cover to provide good concealment which is easily available in the areas surrounding human settlements (Zheng 1987; Lu 1991; Kaledin et al. 2022; Z. Z. Li et al. 2022). The RNP was rarely encountered in Wolong, Tangjiahe, and Heizhugou, where villagers have been evacuated from the NNRs. In Liancheng NNR where human settlements were common, it was symmetrically associated with the *Crossoptilon* (i.e., BEP). Ecological studies show that the RNP and BEP are both found near human settlements, but the BEP feeds on berries, seeds (such as *Potentilla anserina* from the Family Rosaceae and spruce), and vegetable matter (Zheng 2015; Z. Z. Li et al. 2022); and the RNP feeds on matured seeds and agricultural grains (Kaledin et al. 2022). Again, the dietary differentiation supports the symmetric spatial association.

## 5 Conclusion

It is concluded from the above discussions that the diversity of the family Phasianidae may be mainly promoted and maintained by spatial separation. The separation exists extensively at all levels ranging from species to subfamilies. All congeneric species found in this study (i.e., the WEP vs. BEP and the GP vs. LAP) appear in different networks, indicating the separation may be the major driver to speciation of the ancestral populations. The role of separation is further indicated by the monotypicalness (i.e., each of the genera has only one species appearing in a network, although it may contain more species in taxonomy). Spatial association exists in a small proportion of genus pairs. In two of the pairs (i.e., *Ithaginis*‐*Tetraophasis* and *Ithaginis*‐*Crosoptilon*), the genera were asymmetrically associated, which may indicate beneficial interactions and thus minimize competition. The associations in other pairs are symmetric, indicating the genera may compete, but the dietary differentiation alleviates the competition. The phasianid genera maintain biodiversity by space divergence in Palearctic (i.e., Liancheng); whereas in Indomalayan, they do so by evolving differences in ecological features so as to co‐exist in the saturated space. In general, the spatial separation may play a major role in promoting and maintaining the diversity of Phasianidae, supplemented by dietary differentiation and the evolution of beneficial interactions.

## Author Contributions

**Qian Li:** conceptualization (equal), formal analysis (lead), investigation (equal), software (lead), visualization (lead), writing – original draft (lead). **Tengwei Su:** formal analysis (equal), funding acquisition (equal), investigation (equal), resources (equal), software (equal), visualization (equal), writing – original draft (equal). **Yunchuan Zha:** formal analysis (equal), investigation (equal), visualization (supporting), writing – original draft (supporting). **Zhaoyuan Li:** conceptualization (lead), data curation (lead), funding acquisition (lead), methodology (lead), project administration (lead), resources (lead), supervision (lead), validation (lead), writing – review and editing (lead).

## Funding

This study was funded by the State Forestry and Grassland Administration of China, with funds from the International Giant Panda Cooperation Programme (No. 2017115), and the National Natural Science Foundation of China (No. 32171545), Science Foundation of Yunnan Forestry Technological College (Nos. KY(TD)202401 and KY(BS)202401).

## Conflicts of Interest

The authors declare no conflicts of interest.

## Supporting information


**Appendix Table 1.** The 14 phasianids found in Liancheng, Tangjiahe, Wolong, and Heizhugou NNRs in central China from 2017 to 2021.
**Appendix Table 2**. Occurring frequencies (no. camera sites) of phasianids in different habitat dimensions in Liancheng, Tangjiahe, Wolong, and Heizhugou NNRs in central China from 2017 to 2021.
**Appendix Table 3**. Spatial associations of phasianids in Liancheng, Tangjiahe, Wolong, and Heizhugou NNRs in central China from 2017 to 2021.
**Appendix Table 4**. Habitat dimensions that the phasianids co‐occurred in Liancheng, Tangjiahe, Wolong, and Heizhugou NNRs in central China from 2017 to 2021.
**Appendix Table 5**. Phylogenetic distances between the phasianids found in Liancheng, Tangjiahe, Wolong, and Heizhugou NNRs in central China from 2017 to 2021.

## Data Availability

Data files are provided as Supporting Information in the submission system, and all datasets have been permanently archived in Dryad at DOI: 10.5061/dryad.41ns1rnwd.
